# Nanocomposites Based on Pyrolyzed Polyacrylonitrile Doped with FeCoCr/C Transition Metal Alloy Nanoparticles: Synthesis, Structure, and Electromagnetic Properties

**DOI:** 10.3390/polym15173596

**Published:** 2023-08-30

**Authors:** Irina Zaporotskova, Dmitriy Muratov, Lev Kozhitov, Alena Popkova, Natalia Boroznina, Sergey Boroznin, Andrey Vasiliev, Vitaly Tarala, Evgeny Korovin

**Affiliations:** 1 Volgograd State University, Universitetskii Prospect, 100, Volgograd 400062, Russia; boroznina.natalya@volsu.ru (N.B.); boroznin@volsu.ru (S.B.); 2 Institute of Petrochemical Synthesis A.V. Topchiev, Russian Academy of Sciences, Leninsky Prospekt, 29, Moscow 119991, Russia; muratov@misis.ru (D.M.); vasilev.aa@misis.ru (A.V.); 3 National Research Technological University “MISIS”, Leninsky Prospekt, 4, Moscow 119049, Russia; kozitov@rambler.ru; 4 JSC Research Institute of NPO “LUCH”, 24 Zheleznodorozhnaya Str., Podolsk 142103, Russia; popkova-alena@rambler.ru; 5 North Caucasus Federal University, Pushkin Str., 1, Stavropol 355017, Russia; vitaly-tarala@yandex.ru; 6 National Research Tomsk State University, Ave. Lenin, 36, Tomsk 634050, Russia; korovin_ey@mail.tsu.ru

**Keywords:** pyrolyzed polyacrylonitrile, transition metal atoms, nanocomposite, polymers synthesis, FCC lattice, BCC lattice, coherent scattering region, XRF analysis

## Abstract

In the last decade, the development of new materials that absorb electromagnetic radiation (EMR) has received research interest as they can significantly enhance the performance of electronic devices and prevent adverse effects caused by electromagnetic pollution. Electromagnetic radiation absorbers with a low weight and small thickness of the absorber layer, good absorption capacity, and a wide frequency response bandwidth are highly demanded. Here, for the first time, the properties of polymer nanocomposites FeCoCr/C synthesized by doping FeCoCr alloy nanoparticles into a polymer matrix of pyrolyzed polyacrylonitrile are investigated. An analysis of the magnetic properties of FeCoCr/C nanocomposites showed that increasing the synthesis temperature increased the specific magnetization and coercive force values of the FeCoCr/C nanocomposites. The dependence between the ratio of metals in the precursor of pyrolyzed polyacrylonitrile and the electromagnetic and wave-absorbing properties of FeCoCr/C nanocomposites is considered, and the results of complex dielectric and magnetic permeability measurements are analyzed. It is found that the most promising of all the studied materials are those obtained at T = 700 °C with the ratio of metals Fe:Co:Cr = 35:35:30.

## 1. Introduction

While the rapid development of microwave technologies facilitates people’s lives, electromagnetic pollution and absorption in the microwave bandwidth have become a growing concern. In the last decade, there has been a demand for new materials that absorb electromagnetic radiation (EMR), thus improving the reliability of electronic devices and preventing threats to people’s health. The desired electromagnetic radiation absorbers should meet certain requirements, such as low weight and a small thickness of the absorber layer, which allow good absorption capacity and a wide absorption bandwidth.

Carbon-containing materials, due to their chemical stability, low density, adjustable properties, and variety of shapes, are typical components of radio-absorbing materials with dielectric losses and are widely used as the basis for magnetic loss absorbers [1,2,3,4,5,6,7,8,9,10,11,12,13,14,15,16,17,18,19]. However, achieving efficient and broadband absorption, especially with a small thickness of the absorbing layer, is still a challenge. Doping carbon materials with ferromagnetic metal particles seems to solve this problem. By doping magnetic components into carbon-containing materials, composites can be created with higher magnetic losses, improved impedance matching, wide absorption bandwidth, and a high absorption intensity [20,21,22,23,24].

Currently, nanocomposites based on a carbon-containing polymer matrix are receiving much attention from the research community. Polyacrylonitrile (PAN) and its co-polymers, whose structure and composition are determined by synthesis, are among the most effective and promising materials that serve as a base (matrix) for magnetic components, such as iron group metal nanoparticles. PAN contains 68% carbon, and the synthesis parameters provide less weight loss due to the thermal transformations it undergoes during the process. IR heating of PAN allows for obtaining so-called pyrolyzed polyacrylonitrile (PPAN), having in its macromolecules a continuous chain of conjugation of bonds (poly conjugation) [25]. The main benefits of PPAN are low cost, simplicity of synthesis, and the possibility for controlled pyrolysis, which allows for obtaining materials with specified characteristics. The parameters of PPAN-based nanomaterials synthesis allow for controlling the size and shape of nanostructures, resulting in obtaining materials with new functional properties [26,27]. Pyrolyzed polyacrylonitrile can be used to create metal–polymer nanocomposites [28]. In a polymer matrix, soft magnetic nanoparticles can be used to discover new materials.

As a result of the above, today, the urgent issues are the creation of metal composites based on pyrolyzed polyacrylonitrile, used as a polymer matrix for embedded soft magnetic metals, and the study of the mechanisms of interaction between composite and electromagnetic radiation to determine the possible use of the material as a radio-absorbing substance.

Nanoparticles of magnetic metals, such as Fe, Ni, and Co and their alloys with a BCC crystal lattice (for example, Fe-Co), in comparison with ferrites, have higher saturation magnetization and Snook limits, compatible dielectric losses, and magnetic permeability in the gigaHertz frequency range, which makes it possible to increase the efficiency of the material [29,30,31,32,33]. Fe-Co alloy nanoparticles doped into a carbon-containing polymer matrix, which can be designated as FeCo/C, form nanocomposites with enhanced magnetic losses and reduced ability to dielectric losses compared with pure carbon material, which is promising for creating a consistent impedance.

In our research, we developed methods and technology for the synthesis of nanocomposites based on pyrolyzed polyacrylonitrile, containing nanoparticles of various alloys of transition metals in the iron group (Fe-Ni, Fe-Co, Ni-Co) [34,35,36]. It is possible to apply these materials to a wide range of industries: petrochemical synthesis, catalysis, materials of supercapacitor electrodes, gas sensors and electromagnetic radiation insulation, and radio-absorbing materials for microwave electronics devices (provide absorption of microwave radiation up to 80% in the bandwidth of 3–12 GHz and up to 98.7% in the bandwidth of 20–40 GHz).

Our current research focuses on the synthesis of nanocomposites that contain nanoparticles from three or more alloy components; for instance, FeCoAl/C and FeCoNi/C nanocomposites were synthesized [37]. The conditions for the formation of a three-component alloy were found, and the magnetic properties of the obtained nanomaterials were studied. It was shown that the saturation magnetization can reach a value of 140 A m^2^ kg^−1^ with a metal content of no more than 40 wt.%.

In this paper, for the first time, the properties of polymer nanocomposites FeCoCr/C obtained by doping FeCoCr alloy nanoparticles into a polymer matrix of pyrolyzed polyacrylonitrile are synthesized and investigated. Chromium is paramagnetic, but if alloyed, it affects the magnitude of the coercive force, and if chromium is in alloy nanoparticles, it should also influence the anisotropy field. Cobalt in the Fe-Co alloy has the property of increasing the coercive force and saturation induction of the alloy but is an expensive and scarce metal. It is of interest to partially replace it with chromium and evaluate the change in the electrophysical, magnetic, and radio-absorbing properties of the FeCoCr/C.

## 2. Materials and Methods

Synthesis of FeCoCr/C nanocomposites with IR pyrolysis of organometallic precursors was carried out using an automated IR furnace “MILA-5000”(“Ulvac Riko”, Yokohamam, Japan). The MILA-5000 installation is equipped with halogen IR lamps with a total power of 4 kW and a maximum radiation intensity within the range of 0.8 to 1.2 μm. The lamps are installed outside the quartz reactor and are isolated from the reaction zone. This installation is designed to carry out IR heating of samples both in vacuum and in an atmosphere of various gases. In the reaction chamber, temperature control and exposure time are accurate to ±1 °C and ±1 s, respectively. The IR heating temperature is recorded using a chromel–alumel thermocouple placed directly on the sample.

The phase composition and structure of the nanocomposites were determined with X-ray diffraction (XRD) analysis using a powder diffractometer “Difray” 401 (Scientific Instruments, Russia) with Bragg–Brentano focusing (Cr-Kα (wavelength of 0.22909 nm) radiations).

For the synthesis of FeCoCr/C nanocomposites, polyacrylonitrile (PAN) with a molecular weight of 100–250 thousand at. units obtained with redox polymerization were used as an organic base. PAN has many advantages for the synthesis of metal–carbon nanocomposites: due to complexation, it can form solutions with metal compounds of various concentrations and viscosities in polar solvents (dimethyl sulfoxide, nitric acid, dimethylformamide), which allows for a uniform distribution of metal over the volume of the polymer. During the pyrolysis process, PAN is characterized by relatively low mass losses compared with polyvinyl alcohol, polystyrene, and cellulose, due to the fact that the melting point of the polymer is significantly higher than the formation temperature of the cyclic structure of molecules. Therefore, it is used for the synthesis of carbon fibers.

The polymer nanocomposites FeCoCr/C were synthesized from a precursor, which is a system of “polyacrylonitrile–salts of the corresponding metals–solvent dimethylformamide”. Iron acetylacetonate, cobalt acetate, and chromium chloride were used for the synthesis of FeCoCr/C nanocomposites.

A uniform distribution of metal in the polymer was achieved by preparing a solution of PAN and metal salts in dimethylformamide, which was conducted in an oven at a temperature of 50 °C for 4 h. The concentration of PAN in the solution was 5 wt.%, and the total concentration of metals was 20 wt.%. The mass ratio of metals (Fe:Co:Cr) was 40:40:20 and 35:35:30.

The obtained solutions were dried to remove the solvent. Drying was carried out in an oven at temperatures T ≤ 70 °C until the solutions had a constant weight, but the process duration was no longer than 8 h. The drying temperature in the given range did not cause chemical transformation processes in the initial solution. Due to the formation of metal salts and nitrile groups in the polymer complexes, a uniform distribution of metal salts relative to each other and the PAN molecules was maintained, even after the removal of the solvent.

After drying, the solid residue (hereinafter referred to as the precursor) was pyrolyzed in the IR-heated installation. The temperature of IR pyrolysis was controlled using a thermocouple, which was placed directly under the sample and was in contact with it. The heating rate and the duration of the process were also controlled. The process consisted of 2 stages.

Stage 1. Pre-annealing in the air, stepwise: heating to 150 °C with an exposure of 15 min, then heating to 200 °C with an exposure of 15 min. The process was carried out in the air. It is known that under these conditions, the structure of molecules in the polymer (PAN) changes from linear to cyclic with the formation of -C=C- and -C=N- polyconjugation systems. Metal salts decompose to oxides that are no longer coordinated with the nitrile group but with conjugated bonds. Since the process took place in the air, the formation of carbonyl, carboxyl, and hydroxyl groups on PAN molecules was possible.

Stage 2. Heating in vacuum (200 Pa) to a temperature in the range of 500–700 °C with an exposure time of 15 min. The heating rate was 50°/min.

During Stage 1, due to the formation of a developed -C = N- and -C = C- polyconjugation system, a rigid cyclic structure of polymer molecules was created in order to fix metal compounds in this matrix and prevent their diffusion. At the same time, exposure to 200 °C in the air made it possible to convert metal compounds into oxide forms. Heat treatment in the air at 200 °C allowed for accelerating the process of PAN cyclization, which enabled less weight loss during the main stage of IR heating (i.e., during stage 2). The polymer was carbonized and converted into a carbon material (nano-composite matrix). Nanoparticles were obtained simultaneously with the formation of a carbon matrix due to the reduction of metal oxides by PAN pyrolysis products, including H2 and CO. During the course of the reduction, due to partial agglomeration and coalescence, the metals interacted with each other to form nanoparticles of a disordered ternary Fe-Co-Cr solid solution. The type of crystal lattice in such nanoparticles depends on the ratio of metals. If Fe and Cr are excessive in nanoparticles, then BCC lattices are formed, and if Co is excessive, then FCC lattices are formed. In general, there was a correlation between the experiment and the equilibrium diagram for the Fe-Co-Cr system.

In addition to the reduction of metal oxides, the carbon matrix was also reduced. Moreover, during the process of reduction, the detachment of carboxyl and carbonyl groups occurred, and CO was released, which contributed to the restoration of metals.

On the whole, after we cooled down and removed the nanocomposite from the furnace, some of the dangling bonds in the carbon matrix oxidized again when it contacted the air.

The nanoparticles were stabilized in the carbon matrix of the nanocomposite due to Me-C bonds and practically did not undergo secondary oxidation. Large nanoparticles were covered with a graphite shell 5–6 layers thick. This was established in the study of FeCo/C nanocomposites.

The choice of the metal ratio was justified by the presence of solid solutions of various compositions on the state diagrams of the corresponding systems.

X-ray phase and X-ray diffraction studies were performed at room temperature using a DIFRAY X-ray diffractometer with Cr_Kα_ radiation. The results of the experiment were compared with standards from the PDF-4 database (International Centre for Diffraction Data, ICDD). Using the data from the X-ray phase analysis (XPA), calculations for the average sizes of synthesized nanoparticles of the Fe-Co-Cr alloy were carried out according to the Scherer equation [38].

Raman light scattering (RAMAN) spectra were obtained using the inVia Raman Microscope (Renishaw plc) RAMAN spectrometer when excited with a laser with a wavelength of 514 nm. Magnetic measurements were carried out using the advanced vibromagnetometer VSM-100. Measurements for complex values of magnetic and dielectric permittivity were performed with the resonator method using a rectangular multimode resonator. As a microwave generator and indicator, a vector analyzer of circuits E 8363 B from Agilent Technologies was used.

## 3. Results and Discussion

### 3.1. Structure and Composition of Nanocomposites

During the pyrolysis of PAN, a large amount of hydrogen and CO is released. These pyrolysis products are capable of reducing metals from their compounds. At the same time, the process proceeds in the solid phase, which ensures the most effective interaction among the components. In addition, the metals and their compounds can catalyze the process of polymer carbonization. As a result, during the pyrolysis of precursors, metal salts are either hydrolyzed or decomposed to oxides and then reduced to metals by the pyrolysis products of PAN. Since metals are located at a close distance, and the polymer matrix undergoes structural and chemical transformations, metals form nanoparticles of solid solutions.

The doping of chromium into the Fe—Co system, which is often used in the creation of various iron-based magnetic alloys, leads to a change in both the microstructure of the alloy and its properties.

**Figure 1:**
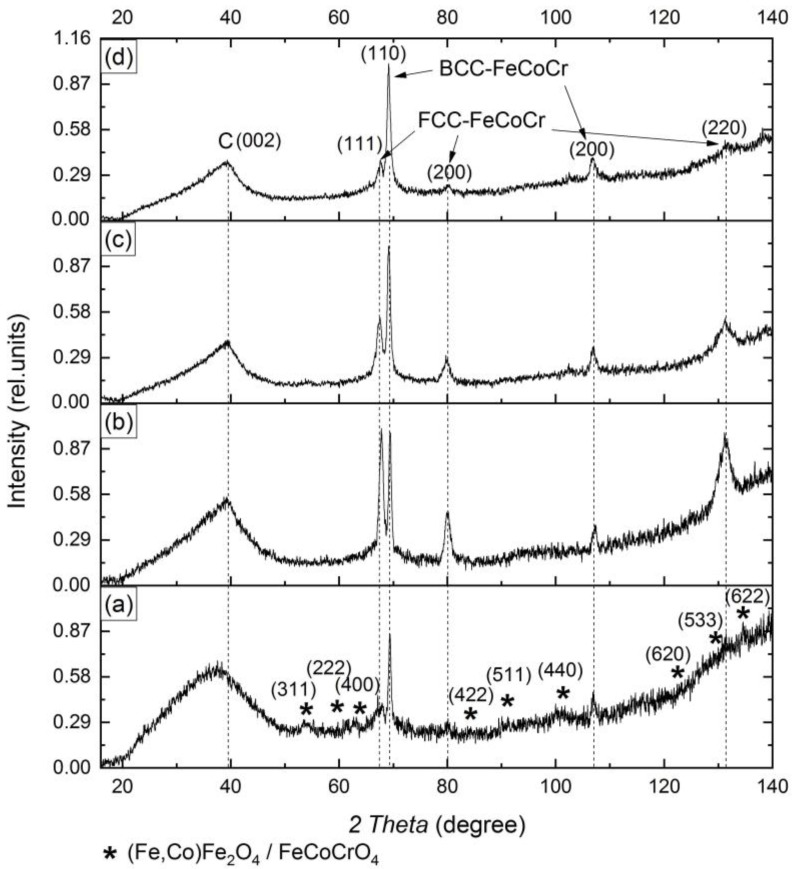
Diffractograms of nanocomposites FeCoCr/C with the ratio of metals Fe:Co:Cr = 40:40:20 synthesized at 500 °C (**a**), 600 °C (**b**), and 700 °C (**c**) and Fe:Co:Cr = 35:35:30 at 700 °C (**d**).

As the results of the XRD analysis show, unlike FeCo/C nanocomposites, the formation of a single-phase system does not occur in PAN-based FeCoCr/C nanocomposites (Figure 1).

According to an analysis of the diffractograms, it can be noted that at a synthesis temperature of 500 °C, nanoparticles with a BCC-type crystal lattice and weak reflexes of the metal phase with a FCC lattice are present. Nanoparticles with a BCC lattice correspond to a solid solution based on Fe-Co. Since the position of the reflexes is shifted relative to the reference, it can be assumed that a triple solid solution of Fe-Co-Cr is formed. Similarly, it can be assumed that the FCC phase is also a triple solid solution based on cobalt with a minimum content of iron and chromium. In addition, there are weak reflexes corresponding to various mixed oxides based on magnetite.

**Figure 2:**
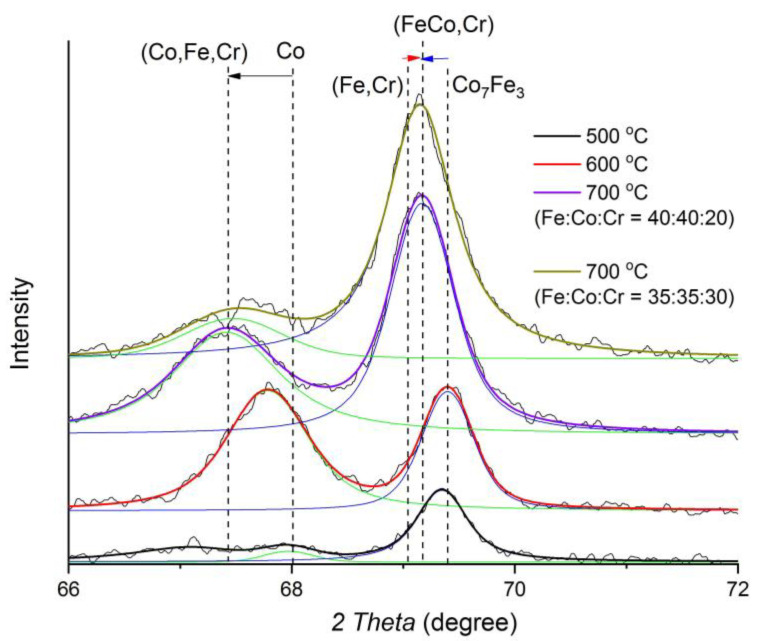
Diffractograms of FeCoCr/C nanocomposites with the ratio of metals Fe:Co:Cr synthesized at different temperatures.

With an increase in the synthesis temperature from 500 to 700 °C, due to the reduction of oxides and coalescence of nanoparticles, a triple solid solution based on the cobalt FCC lattice begins to form. There is a shift in the reflex maxima to the region of small angles, which indicates an increase in the crystal lattice parameter due to the formation of an unordered solid solution of iron and chromium in cobalt (Figure 2). The intensity of the reflexes in the BCC and FCC phases increases, which indicates an increase in the average size of the coherent scattering region (CSR). A comparison of the obtained results with the state diagram for the triple Fe-Co-Cr system [39] showed almost complete correspondence of the phase composition. In the diagram, at the given values for the relative metal content, FCC- and BCC-solid solutions are clearly distinguished.

With a change in the ratio of metals, the basic composition of the identified phases has practically not changed, but there is a change in the ratio of the intensity and areas of the reflexes in the FCC and BCC phases of the Fe-Co-Cr alloy: the proportion of the FCC phase decreases, and the BCC increases. Also, the maximum of the BCC phase reflexes shifts to the left, indicating an increase in the lattice parameter, which is associated with an increase in the proportion of Cr in the corresponding nanoparticles.

**Figure 3:**
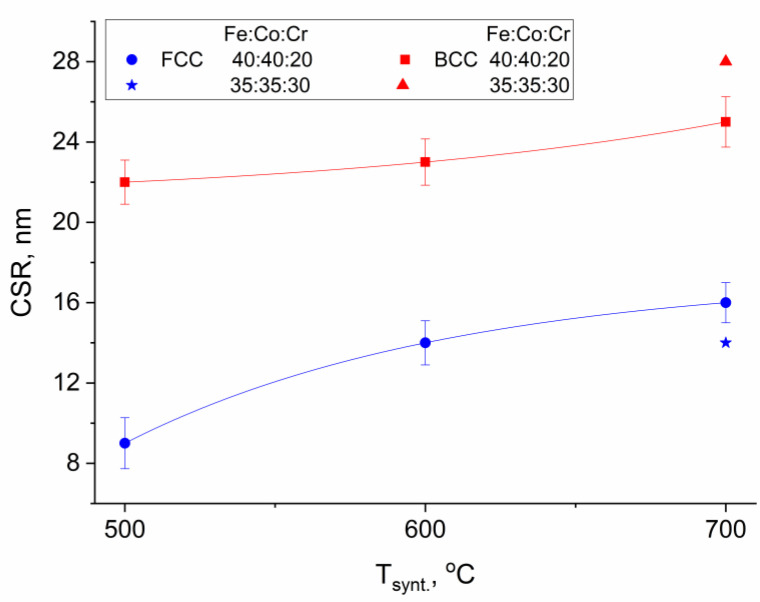
Dependence of the average size of the CSR of Fe-Co-Cr alloy nanoparticles on the synthesis temperature of nanocomposites.

Figure 3 shows the dependence of the average size of the CSR of nanoparticles of alloys with different crystal lattice types on the synthesis temperature.

It was found that the average size of the CSR of the FCC-phase nanoparticles of the alloy varies from 9 to 16 nm, whereas for the BCC phase, it is within the range of 22 to 28 nm, depending on the synthesis temperature and the composition of the nanocomposite.

**Figure 4:**
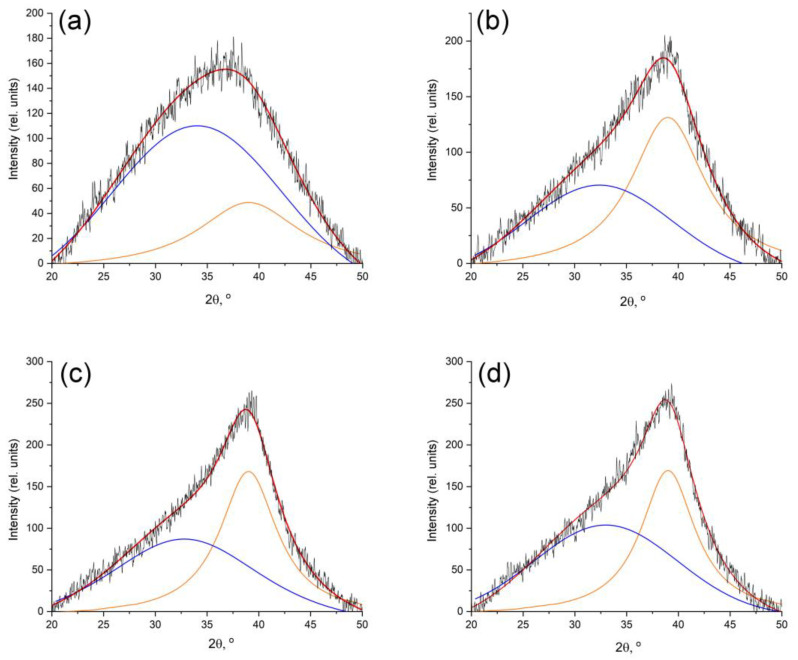
Deconvolution of the peak of the d002 plane—the peak of the carbon matrix of nanocomposites synthesized at different temperatures: (**a**) 500 °C; (**b**) 600 °C; (**c**) 700 °C; (**d**) 700 °C (Fe:Co:Cr = 35:35:30).

In FeCoCr/C nanocomposites, the halo characteristic of carbon materials and nanocomposites based on PAN is fixed on the diffractograms in the area between angles of 20 and 40° (Figure 4).

With an increase in the synthesis temperature from 500 to 600 °C, a clearly pronounced maximum (2θ = 39°) appears, corresponding to the graphite phase. At the same time, the wide halo on the X-ray images is associated with the presence of amorphous carbon, the small size of the coherent scattering regions of the graphite-like phase crystallites, and disturbances in the relative position of the graphene planes of the crystallites relative to each other. The estimation of the average size of the CSR of the graphite-like phase showed an increase in values from 1.3 to 2.1 nm with an increase in the synthesis temperature. An increase in the proportion of chromium in the nanocomposite leads to a decrease in the average size of the CSR to 1.9 nm (Table 1).

Regarding the ratio of the integral areas of the amorphous and graphite-like phases of the carbon-containing polymer matrix of nanocomposites, the dynamics show that with an increase in the synthesis temperature, the proportion of amorphous carbon decreases. However, an increase in the proportion of chromium, all other things being equal, hinders the graphitization process.

**Figure 5:**
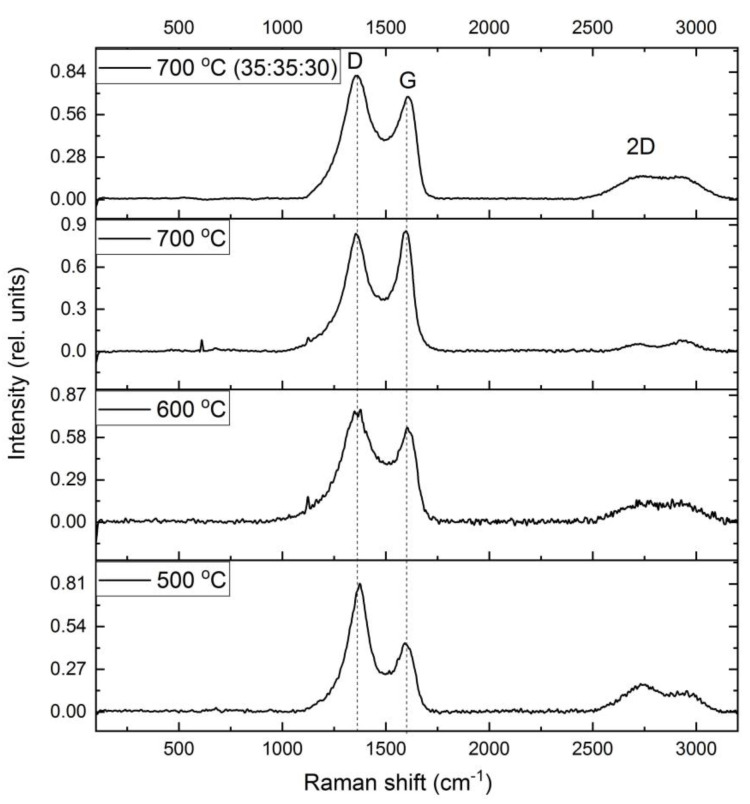
Raman spectra of FeCoCr/C nanocomposites synthesized at different temperatures and metal ratios.

The structural features of the matrix of nanocomposites obtained under various conditions were studied using RAMAN spectroscopy. The results are shown in Figure 5. Clearly defined D- and G-bands are observed in the FeCoCr/C polymer nanocomposites.

It is known that, depending on the microstructure of a carbon-containing material, the position of the maxima of the D- and G-bands change, as well as their intensities in the RAMAN spectrum [40,41,42].

**Figure 6:**
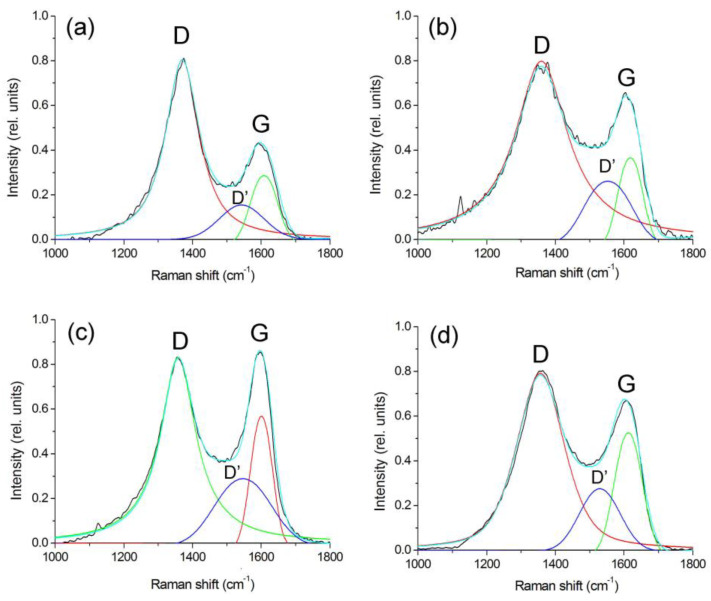
Deconvolution of Raman spectra of FeCoCr/C nanocomposites synthesized at 500 °C (**a**), 600 °C (**b**), 700 °C (**c**), 700 °C (**d**) and the metal ratio Fe:Co:Cr = 35:35:30.

The Raman spectra were analyzed using the method of decomposition into three peaks (Voigt function and Gauss function): D, G, and D’ (Figure 6). The results of the calculations using this technique are presented in Table 2.

Depending on the synthesis conditions, the position of the bands and the ratio of their intensities change. Thus, for samples of nanocomposites synthesized at 500 °C, the intensity ratio (I_D_/I_G_) of the bands is 2.87, and the position of the G-peak corresponds to 1608 sm^−1^. It is known that in nanocrystalline graphite samples, the intensity of the D-peak can exceed the intensity of the G-peak by two times [43]. Consequently, the carbon-containing polymer matrix of PAN nanocomposites FeCoCr/C synthesized at 500 °C has a structure similar to nanocrystalline graphite.

With an increase in the synthesis temperature from 500 to 700 °C, the I_D_/I_G_ ratio tends to 1.47, which indicates an increase in the size of crystallites (La). But in the range of 500–600 °C, a stronger growth is visible, which is associated with the formation of a graphite-like matrix structure from a highly amorphized transition form of carbon. Similar results are shown in the XRD results with respect to the average size of the Lc CSR (it is almost impossible to estimate La using XRD since the corresponding graphite planes give reflexes in the same place as metals). In addition, the diffractograms of nanocomposites synthesized at 600 °C and above show the appearance of a clear maximum at the peak of the plane (002), which is achievable only with relatively large graphite crystallites.

An increase in the proportion of chromium leads to a decrease in the relative intensity of the G-band and an increase in the I_D_/I_G_ ratio to 1.68, i.e., the presence of chromium partially suppresses graphitization.

In addition, there is a change in the intensity of the spectrum in the region of 1520–1570 sm^−1^ (the so-called “saddle”), which usually manifests itself in amorphous carbon in the form of an asymmetric peak due to the fusion of D- and G-bands. With an increase in the synthesis temperature, the intensity of this band decreases with a simultaneous increase in the intensity of the G-band (the I_D’_/I_G_ ratio decreases from 0.56 to 0.49). This trend indicates not only an increase in the size of crystallites but also a decrease in the proportion of amorphous carbon, which correlates with the XRF data. An increase in the relative proportion of chromium from 20 to 30% at the same temperature of synthesis of nanocomposites (700 °C) leads to the formation of smaller graphite crystallites with a higher proportion of amorphous carbon. In general, the results correlate with the XRD data.

Thus, based on the analysis of the RAMAN spectra of metal–carbon polymer composite nanocomposites FeCoCr/C, it was found that with an increase in the synthesis temperature in the matrix microstructure, the size of the clusters in the crystalline component increases by 1.5 to 3 nm and the proportion of disordered (amorphous) carbon decreases.

### 3.2. Magnetic Properties of Nanocomposites

**Figure 7:**
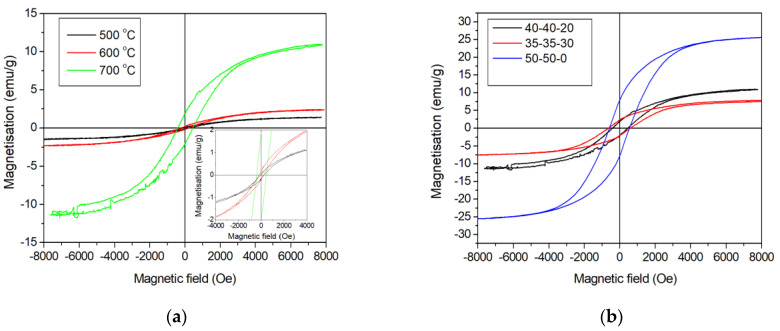
Magnetization reversal loops of FeCoCr/C nanocomposites synthesized at different temperatures (**a**) and metal ratios (**b**).

An analysis of the magnetic properties of nanocomposites (Figure 7) FeCoCr/C showed that with an increase in the synthesis temperature, the values of specific magnetization and coercive force increase (Table 3).

The increase in saturation magnetization from 1.3 to 11.2 A·m^2^·kg^−1^ is determined by the increase in the average size of Fe-Co-Cr alloy nanoparticles in the PPAN polymer matrix at the synthesis temperature range of 500–700 °C. An increase in the relative chromium content from 20 to 30% leads to a decrease in the saturation magnetization from 11.2 to 7.6 A·m^2^·kg^−1^ and an increase in the value of the coercive force. Saturation of nanocomposites synthesized at 700 °C is achieved in fields of ~8 kO.

The increase in saturation magnetization with an increase in the synthesis temperature of nanocomposites is determined by an increase in the average size of the FCC and BCC nanoparticles in the Fe-Co-Cr alloy phases. At synthesis temperatures of 500 °C, superparamagnetic alloy nanoparticles and oxides are present, so the Mr/Ms ratio is small. In addition, the ratio of the size and proportion of nanoparticles significantly influence the saturation magnetization of the FeCoCr/C nanocomposite with different lattice types and compositions.

### 3.3. Radio-Absorbing Properties of FeCoCr/C Nanocomposites

Structural changes in the polymer matrix of nanocomposites, the size, and composition of nanoparticles with an increase in the synthesis temperature cause significant changes in the electromagnetic characteristics of materials.

**Figure 8:**
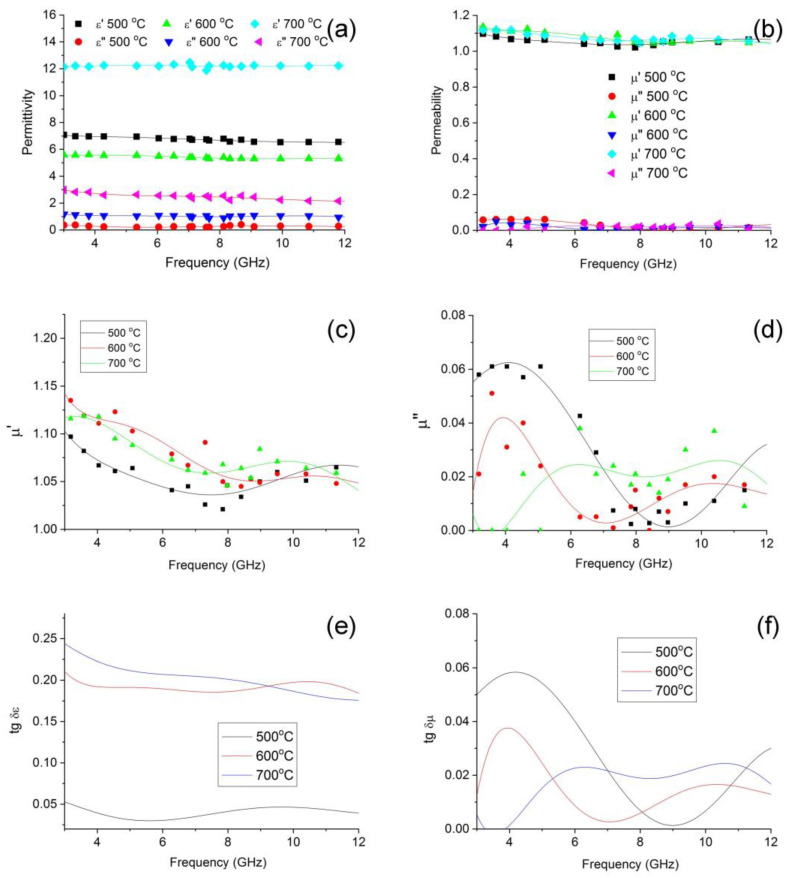
Frequency dependences of complex dielectric (**a**), magnetic (**b**–**d**) permeabilities and tangent of dielectric (**e**) and magnetic (**f**) losses on the synthesis temperature of nanocomposites FeCoCr/C (Fe:Co:Cr = 40:40:20).

The results for the effect of synthesis temperature on the dielectric and magnetic properties of the polymer nanocomposites FeCoCr/C in microwave fields are shown in Figure 8.

When considering the frequency dependencies of the complex permittivity, there is a tendency for both components to increase with an increase in the synthesis temperature. This growth is determined by the formation of a nanoscale heterogeneous structure in the matrix of nanocomposites. It should be noted that the dielectric loss tangent values of FeCoCr/C nanocomposites for the samples synthesized at T = 600 and 700 °C are almost identical, whereas, in the first low-frequency half of the 3–13 GHz band, there is a slight predominance of the sample obtained at 700 °C. That is, the presence of chromium in the precursor causes the formation of metal particles with a composition that initiates the structuring in the carbon-containing polymer matrix. This can also be explained by the formation of thick carbon shells on the nanoparticles. The magnetic loss tangent in the middle of the frequency range under consideration prevails for nanocomposites synthesized at 700 °C, whereas up to 7 GHz, its maximum falls on the FeCoCr/C nanocomposite obtained at 500 °C.

**Figure 9:**
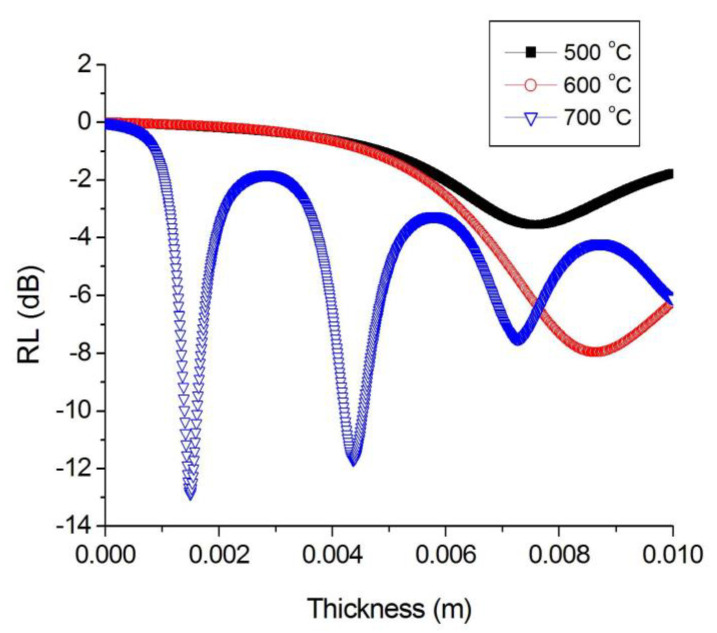
Optimization of the thickness of the absorber layer for nanocomposites synthesized at different temperatures (Fe:Co:Cr = 40:40:20).

The calculations show that the optimal thickness of the absorber layer based on FeCoCr/C nanocomposites differs significantly for samples synthesized at different temperatures. A comparative analysis is presented in Figure 9.

Based on the results, it follows that for samples obtained at 500 and 700 °C, the calculated optimal thickness based on the maximum of the imaginary part of the magnetic permeability is more than 8 mm, which is technologically impractical. However, for the FeCoCr/C 700 °C nanocomposite, several minima are observed including 1.5 mm, 4.3 mm, and 7.3 mm.

**Figure 10:**
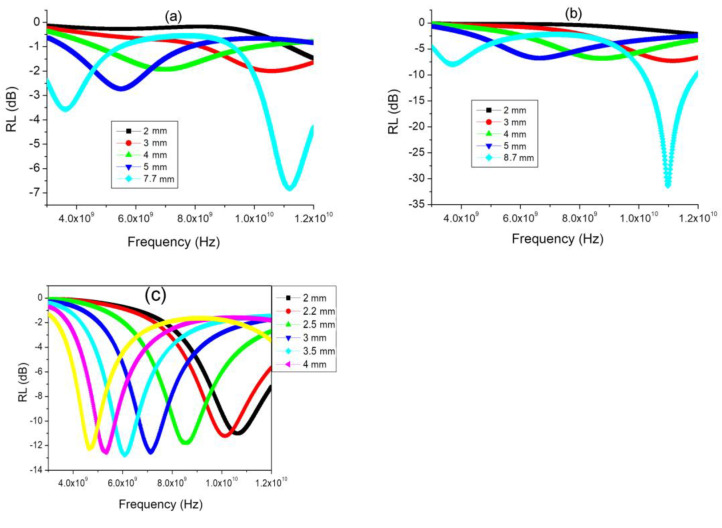
Frequency dependences of the reflection coefficient of FeCoCr/C nanocomposites synthesized at 500 °C (**a**), 600 °C (**b**), and 700 °C (**c**) (Fe:Co:Cr = 40:40:20).

The results of the reflection loss calculations are shown in Figure 10.

It follows from the results that the FeCoCr/C nanocomposite synthesized at 600 °C is effective at a very large thickness. At the same time, the FeCoCr/C nanocomposite obtained at 700 °C with a thickness of 4.3 mm provides an absorption efficiency of 77% at a frequency of 4.9 GHz (RL = −13.8 dB), and only at a thickness of 5 mm, an absorption level of 83% is achieved at a frequency of 12.6 GHz (RL = −15.2 dB).

Thus, the most promising materials are those obtained at T = 700 °C.

### 3.4. Radio-Absorbing Properties of FeCoCr/C Nanocomposites

**Figure 11:**
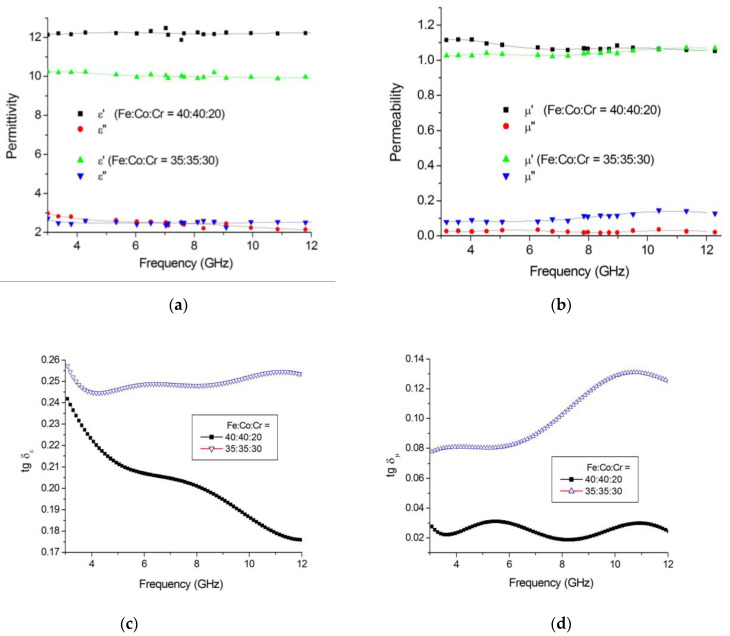
Frequency dependences of the complex dielectric (**a**), magnetic (**b**) permeabilities and the tangent of dielectric (**c**) and magnetic (**d**) losses on the ratio of metals in the precursor (Tsint. = 700 °C).

The effect of the ratio of metals in the precursor of pyrolyzed polyacrylonitrile on the electromagnetic and radio-absorbing properties of FeCoCr/C nanocomposites was considered. The measurement results for the complex dielectric and magnetic permittivity are shown in Figure 11.

The analysis showed that with an increase in the relative Cr content, the tangent of dielectric losses increases, which, apparently, is determined by a change in the electrophysical properties of the alloy nanoparticles and a less pronounced degree of structuring in the carbon matrix. The tangent of magnetic losses also increases from 0.02 to 0.13 rel. units, while the highest growth lies in the region of 6–12 GHz, which is determined by the formation of a predominantly single-phase triple alloy system. That is, instead of two maxima in the imaginary magnetic permeability component corresponding to the presence of nanoparticles with two different types of crystal lattice, we obtain an effect for which the BCC phase of the alloy is mainly responsible. Since the size of the nanoparticles in this phase increased, as well as its relative content in the nanocomposite, a dedicated maximum appears.

**Figure 12:**
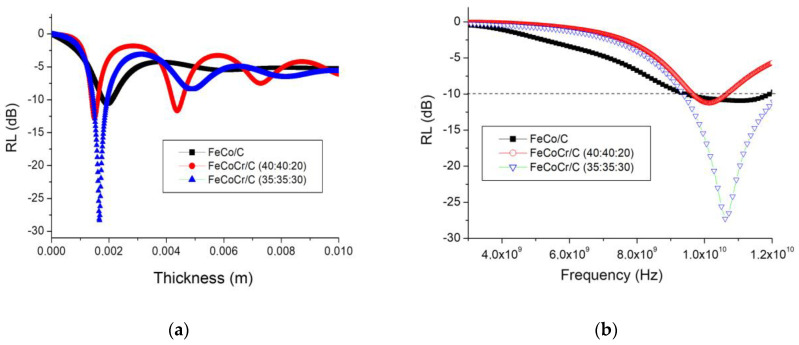
Optimization of the thickness of the absorber layer (**a**) and RL at the optimal thickness (**b**) (Tsint. = 700 °C).

The calculations of the optimal thickness of the absorber layer based on the polymer nanocomposites FeCoCr/C with various compositions (Figure 12) showed that with an increase in the chromium proportion in the nanocomposite from 40-40-20 to 35-35-30, the minimum reflection corresponds to a greater thickness of the material, while the RL value reaches −27 dB. Compared with the nanocomposite FeCo/C, the required thickness is less, while the RL for FeCoCr/C nanocomposites (35:35:30) is significantly lower.

**Figure 13:**
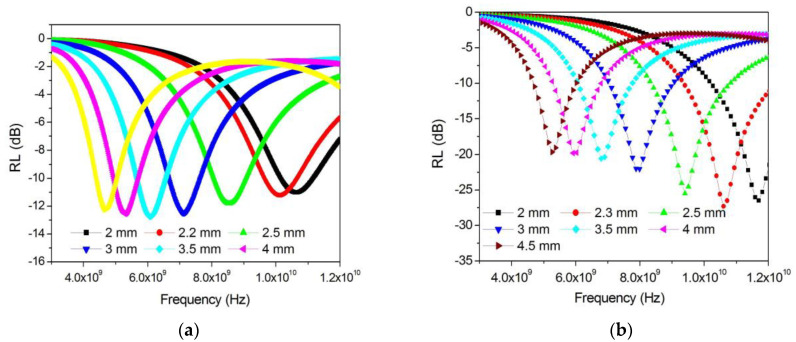
Frequency dependences of the reflection coefficient of nanocomposites (Tsint. = 700 °C) with a different ratio of metals in the precursor for Fe:Co:Cr: 40:40:20 (**a**) and 35:35:30 (**b**).

The results for the calculations of reflection losses for different thicknesses of the absorber layer are shown in Figure 13.

A comparison of the results showed that an increase in the relative chromium content in the nanocomposite makes it possible to obtain an absorption value of 95.7% at a frequency of 10.06 GHz and an absorber layer thickness of 2.3 mm (RL = −27.3 dB). An increase in the layer thickness leads to a shift in the reflection minimum (absorption maximum) to the low-frequency region, while RL increases to −19.7 dB (absorption coefficient decreases to 89.6%) at a frequency of 5.3 GHz and an absorber thickness of 4.5 mm.

Thus, the most promising of all the studied materials are those obtained at T = 700 °C with the ratio of metals Fe:Co:Cr = 35:35:30.

The method utilized in this study allowed us to obtain metal–carbon nanocomposites with the pyrolysis of organometallic precursors formed from a joint solution of a polymer and metal salts. This method is optimal for the following reasons:

- The method is versatile. As a wide range of polymers and metal salts can be used, the main condition is the possibility of joint dissolution in the same solvent.
- The method uses simple equipment. In fact, for complex equipment, only an inert atmosphere furnace is needed.
- The purity of reagents, which are chemically pure.
- The simultaneous formation of nanoparticles and a matrix that stabilizes them but does not prevent the use of materials, for example, in catalysis.
- IR heating for polymers is more efficient than resistive convective heating.
- IR heating has a minimum of inertia as quick heating and cooling can be provided.

However, the method is not free of flaws, which include the following:

- The impossibility of obtaining monodisperse nanoparticles: there will always be a normal-logarithmic distribution.
- The furnace used for IR heating is structurally more complicated, due to the need to use a quartz reactor.
- It is impossible to obtain metal nanoparticles that are strongly oxidized in contact with the air but are reduced at temperatures above 1200 °C.

## 4. Conclusions

This paper carried out studies of nanocomposites based on pyrolyzed polyacrylonitrile acting as a polymer matrix for embedded soft magnetic metals of the iron group. The mechanisms underlying the interaction between such a composite and electromagnetic radiation were also studied to determine the possible use of the material as a radio-absorbing substance.

The properties of FeCoCr/C polymer nanocomposites synthesized by doping Fe-Co-Cr alloy nanoparticles into a polymer matrix of pyrolyzed polyacrylonitrile were investigated for the first time. The choice of this composition of metals was due to the following reasons. Chromium, being paramagnetic, affects the magnitude of the coercive force in alloys, and in alloy nanoparticles, it should also influence the anisotropy field. Cobalt in the Fe-Co alloy has the property of increasing the coercive force and saturation induction of the alloy, but it is an expensive and scarce metal. Therefore, it was of interest to partially replace cobalt with chromium and evaluate the change in the electrophysical, magnetic, and radio-absorbing properties of the FeCoCr/C nanocomposite.

Polymer nanocomposites FeCoCr/C were synthesized from a precursor, which is a system of “polyacrylonitrile–salts of the corresponding metals–solvent dimethylformamide”. The performed X-ray phase and X-ray structural studies of the structural features of polymer nanocomposites obtained under various conditions proved the formation of a system consisting of Fe-Co-Cr alloy nanoparticles embedded in the PPAN polymer matrix. It was found that a rise in the synthesis temperature in the matrix microstructure enabled the size of the clusters in the crystalline component to increase by 1.5 to 3 nm, while the proportion of disordered carbon decreased.

An analysis of the magnetic properties of FeCoCr/C nanocomposites showed that if the synthesis temperature rises, the values of specific magnetization and coercive force increase. The increase in saturation magnetization at rising synthesis temperatures of nanocomposites is determined by an increase in the average size of the FCC and BCC nanoparticles of the FeCoCr alloy phases in the polymer matrix. In addition, the saturation magnetization of the FeCoCr/C nanocomposite is significantly influenced by the ratio of sizes and fractions of nanoparticles with different lattice types and compositions.

It was established that structural changes in the polymer matrix of nanocomposites and changes in the size and composition of nanoparticles with an increase in the synthesis temperature cause significant changes in the electromagnetic characteristics of materials. The effect of the synthesis temperature on the dielectric and magnetic properties of polymer nanocomposites FeCoCr/C was investigated in the microwave bandwidth. It was shown that the optimal thickness of the absorber layer based on FeCoCr/C nanocomposites differs significantly for samples synthesized at different temperatures, while the most promising materials are those obtained at T = 700 °C.

The influence of the ratio of metals in the precursor of pyrolyzed polyacrylonitrile on the electromagnetic and radio-absorbing properties of FeCoCr/C nanocomposites was considered. The results for the measurements of complex dielectric and magnetic permeability are analyzed. It was proven that the most promising of all studied materials are those obtained at T = 700 °C with the ratio of metals Fe:Co:Cr = 35:35:30.

## Figures and Tables

**Table 1:** Characteristics of nanocomposites.

T_synth._, °C	Ratio Fe:Co:Cr	Phases FeCoCr	CSR _np_^1^, nm	CSR m ^2^, nm	A(C_am_)/A(C_g_) ^3^
500	40:40:20	FCC BCC	9 22	1.3	3.4
600		FCC BCC	14 23	1.8	1.3
700		FCC BCC	16 24	2.6	1.1
700	35:35:30	FCC BCC	12 28	2.2	1.2

^1^ CSR _np_—the average size of the CSR of FeCoCr nanoparticles. ^2^ CSR _m_ is the average size of the CSR of a graphite-like polymer matrix of nanocomposites. ^3^ A(C_am_)/A(C_g_) is the ratio of the peak areas in amorphous (C_am_) and graphite-like (C_g_) carbon.

**Table 2:** Characteristics of nanocomposites.

T_synth._, °C	Fe:Co:Cr	ν(I_D_), sm^−1^	ν(I_G_), sm^−1^	ν(I_D’_), sm^−1^	I_D_/I_G_	I_D’_/I_G_	L_a_, nm
500	40:40:20	1370	1608	1531	2.87	0.56	1.5
600		1359	1618	1553	1.77	0.53	2.5
700		1358	1601	1547	1.47	0.49	3.0
700	35:35:30	1356	1612	1530	1.68	0.52	2.6

**Table 3:** Magnetic characteristics of FeCoCr/C nanocomposites.

T_synth._, °C	Fe:Co:Cr	M_s_, A·m^2^·kg^−1^	M_r_, A·m^2^·kg^−1^	H_c_, O	M_r_/M_s_	D_np_, nm (FCC/BCC)
500	40:40:20	1.3	0.05	168	0.04	9/22
600		2.5	0.18	205	0.07	14/23
700		11.2	1.89	424	0.17	16/24
700	35:35:30	7.6	2.4	620	0.32	12/28
700	50:50:0	25.8	7.8	562	0.30	0/14

## Data Availability

Not applicable.

## References

[1] Qiang R., Du Y.C., Wang Y., Wang N., Tian C.H., Ma J., Xu P., Han X.J. (2016). Rational design of yolk-shell C@C microspheres for the effective enhancement in microwave absorption. Carbon.

[2] Zhang Y., Huang Y., Chen H.H., Huang Z.Y., Yang Y., Xiao P.S., Zhou Y., Chen Y.S. (2016). Composition and structure control of ultralight graphene foam for high-performance microwave absorption. Carbon.

[3] Zhu H.P., Zhang H.Y., Chen Y.M., Li Z.H., Zhang D.F., Zeng G.X., Huang Y.X., Wang W.G., Wu Q.B., Zhi C.Y. (2016). The electromagnetic property and microwave absorption of wormhole-like mesoporous carbons with different surface areas. J. Mater. Sci..

[4] Lu M.M., Cao M.S., Chen Y.H., Cao W.Q., Liu J., Shi H.L., Zhang D.Q., Wang W.Z., Yuan J. (2015). Multiscale assembly of grapelike ferroferric oxide and carbon nanotubes: A smart absorber prototype varying temperature to tune intensities. ACS Appl. Mater. Interfaces.

[5] Xiang J., Li J.L., Zhang X.H., Ye Q., Xu J.H., Shen X.Q. (2014). Magnetic carbon nanofibers containing uniformly dispersed Fe/Co/Ni nanoparticles as stable and high-performance electromagnetic wave absorbers. J. Mater. Chem. A.

[6] Lv H.L., Ji G.B., Liu W., Zhang H.Q., Du Y.W. (2015). Achieving hierarchical hollow carbon@Fe@Fe304 nanospheres with superior microwave absorption properties and lightweight features. J. Mater. Chem. C.

[7] Wang L.N., Xia X.L., Li Y.F., Yang F., Zhang L.Q., Liu L.P., Ren X., Yang H.T. (2014). Synthesis and microwave absorption property of flexible magnetic film based on graphene oxide/carbon nanotubes and Fe304 nanoparticles. J. Mater. Chem. A.

[8] Yuan K.P., Che R.C., Cao Q., Sun Z.K., Yue Q., Deng Y.H. (2015). Designed fabrication and characterization of three-dimensionally ordered arrays of core-shell magnetic mesoporous carbon microspheres. ACS Appl. Mater. Interfaces.

[9] Li G.M., Wang L.C., Li W.X., Xu Y. (2015). Fe-, Co-, and Ni- loaded porous activated carbon balls as lightweight microwave absorbents. ChemPhysChem.

[10] He P., Hou Z.L., Zhang K.L., Li J., Yin K., Feng S., Bi S. (2017). Lightweight ferroferric oxide nanotubes with natural resonance property and design for broadband microwave absorption. J. Mater. Sci..

[11] Lv H., Zhihong Y., Hongge P. (2022). Electromagnetic absorption materials: Current progress and new frontiers. Prog. Mater. Sci..

[12] Feng W., Liu Y., Bi Y., Su X., Lu C., Han X., Ma Y., Feng C., Ma M. (2023). Recent advancement of magnetic MOF composites in microwave absorption. Synth. Met..

[13] Wu D., Wang Y., Deng S., Lan D., Xiang Z., He Q. (2023). Heterostructured CoFe@ N-doped carbon porous polyhedron for efficient microwave absorption. Nano Res..

[14] Gai L., Zhao H., Wang F., Wang P., Liu Y., Han X., Du Y. (2022). Advances in core—Shell engineering of carbon-based composites for electromagnetic wave absorption. Nano Res..

[15] Ma M., Liao Z., Su X., Zheng Q., Liu Y., Wang Y., Ma Y., Wan F. (2022). Magnetic CoNi alloy particles embedded N-doped carbon fibers with polypyrrole for excellent electromagnetic wave absorption. J. Colloid Interface Sci..

[16] Wu Y., Peng K., Man Z., Zang R., Li P., Liu S., Wang S., Liu P., Li P., Cui Y. (2022). A hierarchically three-dimensional CoNi/N-doped porous carbon nanosheets with high performance of electromagnetic wave absorption. Carbon.

[17] Ajia S., Asa H., Toyoda Y., Sato M., Matsuura M., Tezuka N., Sugimoto S. (2022). Development of an alternative approach for electromagnetic wave absorbers using Fe–Cr–Co alloy powders. J. Alloys Compd..

[18] Sun L., Jia Z., Xu S., Ling M., Hu D., Liu X., Zhang C., Wu G. (2022). Synthesis of NiCo_2_-0.5 xCr_2_O_3_@ C nanoparticles based on hydroxide with the heterogeneous interface for excellent electromagnetic wave absorption properties. Compos. Commun..

[19] Dai B., Ma Y., Dong F., Yu J., Ma M., Thabet H.K., El-Bahy S.M., Ibrahim M.M., Huang M., Seok I. (2022). Overview of MXene and conducting polymer matrix composites for electromagnetic wave absorption. Adv. Compos. Hybrid Mater..

[20] Qiang R., Du Y.C., Zhao H.T., Wang Y., Tian C.H., Li Z.G., Han X.J., Xu P. (2015). Metal organic framework-derived Fe/C nanocubes toward efficient microwave absorption. J. Mater. Chem. A.

[21] Wang J.C., Zhou H., Zhuang J.D., Liu Q. (2015). Magnetic gamma-Fe203, Fe304, and Fe nanoparticles confined within ordered mesoporous carbons as efficient microwave absorbers. Phys. Chem. Chem. Phys..

[22] Wu G.L., Cheng Y.H., Ren Y.Y., Wang Y.Q., Wang Z.D., Wu H.J. (2015). Synthesis and characterization of c-Fe203@C nanorod-carbon sphere composite and its application as microwave absorbing material. J. Alloys Compd..

[23] Yang H., Wang A., Feng X., Dong H., Zhuang T., Sui J., Zhao S., Sun C. (2023). PPyNT/NR/NBR Composites with Excellent Microwave Absorbing Performance in X-Band. Polymers.

[24] Liu P., Ming V., Ng H., Yao Z., Zhou J., Lei Y., Yang Z., Lv H., Bing L. (2017). Facile synthesis and hierarchical assembly of flowerlike NiO structures with enhanced dielectric and microwave absorption properties. ACS Appl. Mater. Interfaces.

[25] Berlin A., Geyderikh M., Davydov B., Kargin V., Karpacheva G., Krenzel B., Khukhareva G. (1972). Chemistry of Polyconjoint Systems.

[26] Liu M., Wu L., Fan B., Tong G., Chen D.B., Wu W. (2022). Governing the Ni content and size of 2D layered C/Ni nanoparticle composites for enhanced electromagnetic wave absorption. Appl. Surf. Sci..

[27] Ren X., Gao Z., Wu G. (2022). Tunable nano-effect of Cu clusters derived from MOF-on-MOF hybrids for electromagnetic wave absorption. Compos. Commun..

[28] Ghorpade R.V., Cho D.W., Hong S.C. (2017). Effect of controlled tacticity of polyacrylonitrile (co)polymers on their thermal oxidative stabilization behaviors and the properties of resulting carbon films. Carbon.

[29] Li G.X., Guo Y.X., Sun X., Wang T., Zhou J.H., He J.P. (2012). Synthesis and microwave absorbing properties of FeNi alloy incorporated ordered mesoporous carbon-silica nanocomposite. J. Phys. Chem. Solids.

[30] Han Z., Li D., Wang H., Liu X.G., Geng D.Y., Zhang Z.D. (2009). Broadband electromagnetic-wave absorption by FeCo/C nanocapsules. Appl. Phys. Lett..

[31] Kodama D., Shinoda K., Kasuya R., Doi M., Tohji K., Jeyadevan B. (2012). Potential of sub-micron-sized Fe-Co particles for antenna applications. J. Appl. Phys..

[32] Yu Z.X., Zhang N., Yao Z.P., Han X.J., Jiang Z.H. (2013). Synthesis of hierarchical dendritic micro-nano structure Cox-Fel-x alloy with tunable electromagnetic absorption performance. J. Mater. Chem. A.

[33] Wang X.Q., Liang C.D., Dai S. (2008). Facile synthesis of ordered mesoporous carbons with high thermal stability by self-assembly of resorcinol-formaldehyde and block copolymers under highly acidic conditions. Langmiur.

[34] Karpenkov D.Y.u., Kozitov L.V., Skokov K.P., Karpenkov A.Y.u., Popkova A.V., Gutfleisch O. (2017). Infrared heating mediated synthesis and characterization of FeCo/C nanocomposites. J. Magn. Magn. Mater..

[35] Kozitov L.V., Muratov D.G., Kostishin V.G., Suslyaev V.I., Korovin E.Y.U., Popkova A.V. (2017). FeCo/C Nanocomposites: Synthesis, Magnetic and Electromagnetic Properties. Russ. J. Inorg. Chem..

[36] Yakushko E.V., Kozhitov L.V., Muratov D.G., Kostishin V.G. (2016). Synthesis and magnetic properties of NiCo/C nanocomposites. J. Inorg. Chem..

[37] Muratov D.G., Kozhitov L.V., Emelyanov S.G., Vasilyev A.V., Popkova A.V. (2016). The syntesis of nanoparticles of ternary alloys Fe-Co-Ni encapsulated in the carbon matrix of nanocomposites Fe-Co-Ni/C. J. Nano-Electron. Phys..

[38] Patterson A. (1939). The Scherrer Formula for X-Ray Particle Size Determination. Phys. Rev. J..

[39] Bannykh O., Budberg P., Alisova S. (1986). Diagrams of the State of Double and Multicomponent Systems Based on Iron.

[40] Ferrari A., Robertson J. (2000). Interpretation of Raman spectra of disordered and amorphous carbon. Phys. Rev. B Condens. Matter Mater. Phys..

[41] Ferrari A.C. (2007). Raman spectroscopy of graphene and graphite: Disorder, electron-phonon coupling, doping and nonadiabatic effects. Solid State Commun..

[42] Tunistra F., Koenig J. (1970). Raman spectrum of graphite. J. Chem. Phys..

[43] Simionescu O.G., Popa R.C., Avram A., Dinescu G. (2020). Thin films of nanocrystalline graphene/graphite: An overview of synthesis and applications. Plasma Process. Polym..

